# Prevalence and Molecular Characterisation of Extended-Spectrum Beta-Lactamase-Producing Shiga Toxin-Producing *Escherichia coli*, from Cattle Farm to Aquatic Environments

**DOI:** 10.3390/pathogens11060674

**Published:** 2022-06-10

**Authors:** Khuliso Ramaite, Mutshiene Deogratias Ekwanzala, Maggy Ndombo Benteke Momba

**Affiliations:** 1 Department of Environmental, Water and Earth Sciences, Tshwane University of Technology, Pretoria 0001, South Africa; 211148365@tut4life.ac.za (K.R.); ekwanzalamd@tut.ac.za (M.D.E.); 2 Centre for Antibiotic Resistance Research (CARe), University of Gothenburg, 41346 Gothenburg, Sweden; 3 Department of Infectious Diseases, Institute of Biomedicine, University of Gothenburg, 41346 Gothenburg, Sweden

**Keywords:** extended-spectrum β-lactamases, Shiga toxin-producing *Escherichia coli*, husbandry soil, manure, water, livestock

## Abstract

Extended-spectrum beta-lactamase (ESBL)-producing bacteria are a major problem for public health worldwide because of limited treatment options. Currently, only limited information is available on ESBL-producing Shiga toxin-producing *Escherichia coli* (STEC) in cattle farms and the surrounding aquatic environment. This study sought to track and characterise ESBL-producing STEC disseminating from a cattle farm into the water environment. Animal husbandry soil (HS), animal manure (AM), animal drinking water (ADW), and nearby river water (NRW) samples were collected from the cattle farm. Presumptive ESBL-producing STEC were isolated and identified using chromogenic media and mass spectrophotometry methods (MALDI-TOF-MS), respectively. The isolates were subjected to molecular analysis, and all confirmed ESBL-producing STEC isolates were serotyped for their O serogroups and assessed for antibiotic resistance genes (ARGs) and for the presence of selected virulence factors (VFs). A phylogenetic tree based on the multilocus sequences was constructed to determine the relatedness among isolates of ESBL-producing STEC. The highest prevalence of ESBL-producing STEC of 83.33% was observed in HS, followed by ADW with 75%, NRW with 68.75%, and the lowest was observed in AM with 64.58%. Out of 40 randomly selected isolates, 88% (*n* = 35) belonged to the serogroup O45 and 13% (*n* = 5) to the serogroup O145. The multilocus sequence typing (MLST) analysis revealed four different sequence types (STs), namely ST10, ST23, ST165, and ST117, and the predominant ST was found to be ST10. All 40 isolates carried sul1 (100%), while *bla*_OXA_, *bla*_CTX-M_, *sul*2, *bla*_TEM_, and *qnrS* genes were found in 98%, 93%, 90%, 83%, and 23% of the 40 isolates, respectively. For VFs, only stx2 was detected in ESBL-producing STEC isolates. The results of the present study indicated that a cattle environment is a potential reservoir of ESBL-producing STEC, which may disseminate into the aquatic environment through agricultural runoff, thus polluting water sources. Therefore, continual surveillance of ESBL-producing STEC non-O157 would be beneficial for controlling and preventing STEC-related illnesses originating from livestock environments.

## 1. Introduction

The availability of freshwater is a challenge worldwide [1,2]. In developing countries, including South Africa, the majority of people living in non-metropolitan areas still use and depend on untreated surface water sources such as rivers, dams, and lakes and are at risk of waterborne diseases as there is limited access to treated water [3,4,5]. It has been estimated that 3.8 million people, especially inhabitants of informal settlements in South Africa, depend on polluted untreated water or groundwater due to the lack of access to communal improved water sources [4]. However, the aquatic environments are known to harbour and disseminate antibiotic resistance (AR), and human health is at risk when they are exposed to antibiotic-resistant bacteria (ARB) [6]. The death toll of infections caused by AR is at least 33,000 each year, in Europe alone [7].

In South Africa, approximately 70% of the agricultural land is used for livestock farming [8]. Antibiotics in livestock farms are mainly used for treatment, prevention of diseases, and as growth promoters to address the demand for food [9]. Inappropriate use of antibiotics in livestock has resulted in the development of AR worldwide [10]. In food animals such as cattle, chicken, and pigs, the use of antibiotics is expected to rise by 67% in BRICS countries (Brazil, Russia, India, China, and South Africa) by 2030 [9,11]. About 20% to 80% of the antibiotics ingested by livestock are excreted into the environment and persist there [12]. Excessive use of antibiotics in livestock has also led to the emergence of ARB and ARGs [8]. Humans can be exposed to ARB and ARGs through contaminated food, direct contact with infected animals, and by exposure to the contaminated environment [13].

Several environmental ARBs and ARGs resistomes are considered as the hotspots for AR. These environments include manure; soils; and aquatic environments such as rivers, lakes, and streams [14]. Hence, water pollution is a major challenge worldwide, and agriculture is one of the main contributors [15]. There is evidence that antibiotics have emerged as agricultural pollutants originating from veterinary medicines disseminating from farms into water sources [15]. Consequently, it is imperative to investigate the presence of ARB and its associated ARGs in livestock farms disseminating into the nearby water sources.

The World Health Organization (WHO) published for the very first time a list of antibiotic-resistant priority pathogens that pose the greatest threat to human health [16]. Among the reported threats, Enterobacteriaceae, which include *E. coli*, were listed as one of the most critical groups of multidrug-resistant bacteria posing treatment failures and threats in hospitals. The ESBL-producing Enterobacteriaceae cause considerable morbidity in the community [16]. *E. coli* is known to cause infections in humans and is commonly used as an indicator of faecal contamination in the environments, especially in aquatic systems [17,18]. Certain strains of *E. coli* are responsible for diarrhoea in humans; these include verocytotoxigenic *E. coli* (VTEC) strains, which have been recognised as the cause of mild to severe diarrhoea in humans, haemorrhagic colitis, and haemolytic uremic syndrome [19]. Shiga toxin-producing *E. coli* (STEC) may also be referred to as VTEC [20]. They are an emerging zoonotic foodborne and waterborne pathogen that causes serious health complications in humans and are an important cause of foodborne outbreaks [21,22].

Humans can acquire STEC through consumption of contaminated food or water, contact with infected animals, and exposure to a contaminated environment [23,24]. Over 600 serogroups of STEC have been identified globally from different foods, humans, other animals, and the environment [22]. Ruminants, particularly cattle, are considered as the primary reservoirs of STEC and major reservoirs of O157 and non-O157 STEC [23,25]. These pathogens do not cause disease to the host, but colonise the stomach lining and are shed through faeces [25]. *E. coli* O157:H7 is a notable STEC strain that has been associated with cattle [26]. In South Africa, *E. coli* O157:H7 and non-O157 have been previously identified from livestock and humans faecal samples, meat products, and water samples [27]. However, STEC serogroups have not been identified in cattle environment matrices (HS, AM, and ADW) that potentially disseminate these pathogens into aquatic environments.

Per year, it has been estimated that STEC accounts for 2.8 million cases of acute human diseases worldwide, and causes 3890 cases of the haemolytic uremic syndrome (HUS) and 230 deaths [19,28]. In Africa, 10,200 cases of STEC infection occur each year [19]. The first case of STEC in South Africa was reported in 1990, and an outbreak in 1992 caused by STEC was reported after consumption of water contaminated by cattle carcasses [19,24].

In clinical settings, serogroup O104:H4 *E. coli* was identified from over 4000 suspected diarrhoeagenic cases isolated between 2004 and 2011 [29]. Smith et al. [30] reported STEC serotype O26:H11 from human stools. Shiga toxin-producing *E. coli* isolates were implicated in human disease outbreaks between 2006 and 2013, which were fully serotyped and identified as STEC O26:H11, O111:H8, O157:H7, and O117:H7 serotypes [22].

Multilocus sequence typing (MLST) is used for typing pathogenic *E. coli* strains to establish the evolutionary relationship and relatedness between isolates [31]. For environmental samples, MLST has been previously employed to investigate the circulating sequence in environmental water sources [32]. For the identified and characterised O157:H7, sequence types (STs—ST10, ST11, and ST1204) were assigned from rivers and runoff water [32]. ST131 has been described to be caused by ESBL-producing *E. coli* from the community in hospitalised patients of South Africa [33] and in ESBL-producing *E. coli* isolates; ST131 clone was also identified from the community in hospital patients [34]. The predominant sequence type of ESBL-producing STEC circulating in the cattle environment has not yet been compared to those present in an adjacent aquatic environment.

Extended-spectrum β-lactamase Enterobacteriaceae are a major public health threat [35]. It has been reported that in developing countries, the spread and burden of ESBL-producing bacteria is high [36]. *E. coli* is one of the major pathogens of ESBL that causes complicated urinary tract infections (UTI), and poses significant treatment failure against cephalosporins [36]. Also, ESBL-producing *E. coli* is considered as the cause of community-acquired infection [37]. According to a review by Ekwanzala et al. [14], various multidrug resistance genes and ESBL resistance genes of *E. coli* from clinical, environmental, and farm settings have been identified and detected in cattle manure samples [38]. A few studies have characterised ARGs of ESBL-producing STEC in cattle manure. However, no study has focused on the detection of ARGs that are present in the cattle environment. Therefore, this study sought to investigate the presence of ARGs among ESBL-producing STEC in cattle-associated environments, including aquatic environments.

In livestock farms, VFs have been previously detected in faecal samples of animal origin [12,23,27,39,40]. Shiga toxin type 1 (*Stx*1) and type 2 (*Stx*2), encoded by *stx*1 and *stx*2 genes, are the major VFs of STEC [41]. Virulence genes belonging to STEC serogroups have not been assessed in a cattle environment. Although several studies have investigated STEC and ESBL-producing *E. coli* from cattle manure, there is no information on extended-spectrum beta-lactamases (ESBLs) among the STEC serogroups, particularly in a contaminated cattle environment. The current study, therefore, investigated the prevalence of and characterised the ESBL-producing STEC in cattle-associated environments by serotyping the STEC serogroups; typing STs; and detecting selected ARGs and the virulence genes harboured in HS, AM, ADW, and NRW samples, by using MLST-based phylogenetic analysis to establish relatedness.

## 2. Results

### 2.1. Prevalence of Extended-Spectrum Beta-Lactamase-Producing Shiga Toxin-Producing Escherichia coli

**Figure 1:**
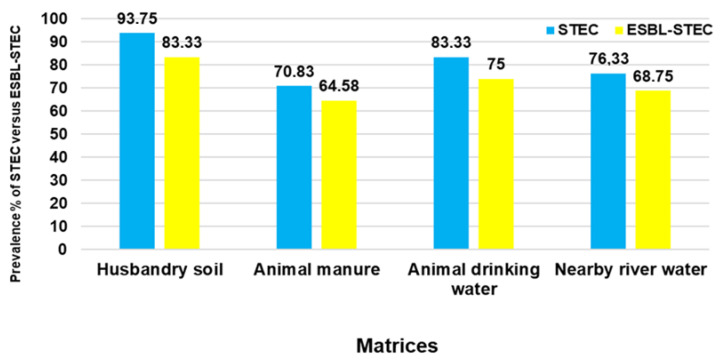
The prevalence of STEC and ESBL-producing STEC culture-positive samples per sample matrix.

Using CHROMagar™ STEC media, it was found that 83% (*n* = 158) of the 192 samples representing all the matrices carried presumptive STEC colonies. Animal husbandry soil harboured the highest percentage of STEC at 94% (*n* = 45/48), followed by ADW at 83% (*n* = 40/48), NRW at 79% (*n* = 38/48), and AM at 71% (*n* = 34/48). In all the matrices, 73.95% (*n* = 142) of the samples were found to carry presumptive ESBL-producing STEC colonies using CHROMagar™ STEC supplemented with CHROMagar™ ESBL supplement. The presumptive prevalence of ESBL-producing STEC was found to be the highest in HS at 83% (*n* = 40/48), followed by 75% (*n* = 36/48) in ADW, 69% (*n* = 33/48) in NRW, and the lowest prevalence was found in AM at 65% (*n* = 31/48). The prevalence of culture-positive samples for STEC was compared to the positive samples for ESBL-producing STEC per matrix for a simplified interpretation of the data, as illustrated in Figure 1. All the presumptive isolates (100 in total and 25 from each matrix) were confirmed as *E. coli*. Of all the 100 confirmed ESBL-producing STEC, 40 isolates were randomly selected (10 from each matrix) for the MLST phylogenetic analysis, and for the detection of virulence factor genes and ARGs.

### 2.2. Serogroups of ESBL-Producing STEC

The PCR results of the selected isolates revealed two serogroups, namely O45, with 88% (*n* = 35), and O145, with 13% (*n* = 5). For the 10 randomly selected isolates from each matrix (HS, AM, and ADW), 9 isolates from each matrix were identified as O45 and 1 isolate as O145. In NRW, 8 isolates were identified as O45 and 2 isolates as O145.

### 2.3. Multilocus Sequence Typing Profiles

**Figure 2:**
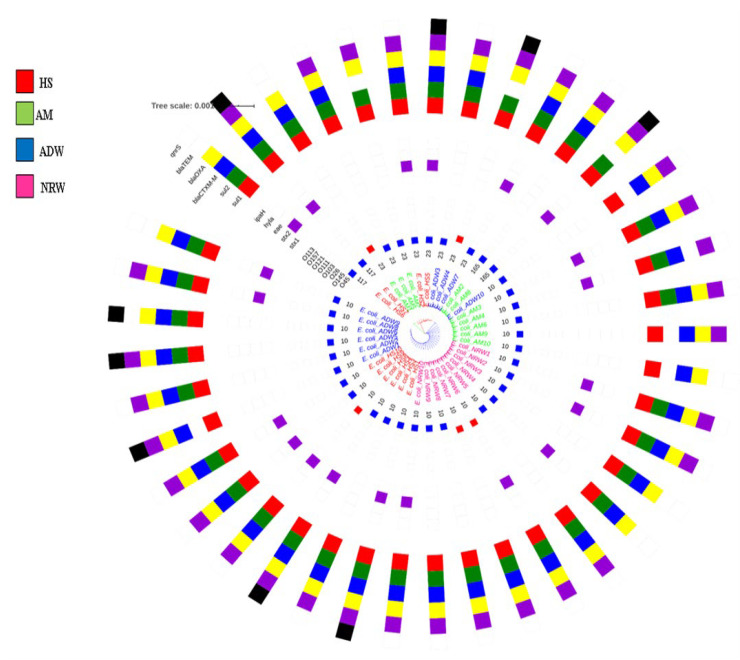
MLST-based dendrogram of *E. coli* isolates from HS, AM, ADW, and NRW highlighting selected STs, serogroups, virulence factor genes, and ARGs.

**Figure 3:**
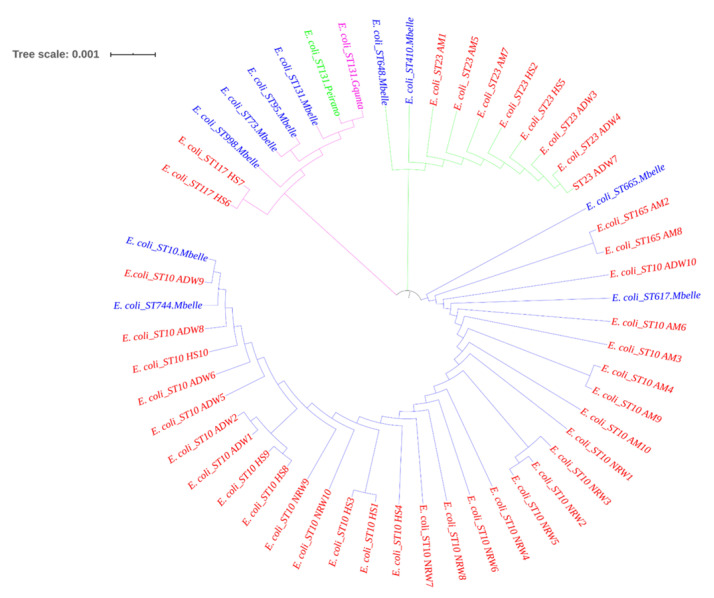
Phylogenetic tree of *E. coli* isolates generated using iTol v.3. The isolates from this study are indicated in red; the other South African clinical isolates are in blue.

A total of 40 isolates were investigated by MLST analysis, which revealed four different sequence types. The predominant sequence type was ST10, which was represented by 28 isolates. The remaining isolates were assigned to three unique STs, namely ST23, ST117, and ST165 for eight, two, and two isolates, respectively. The maximum likelihood approach based on the Tamura–Nei model showed seven (7) concatenated housekeeping gene sequences (Figure 2). The tree with the highest log likelihood (−5248.14) is shown in Figure 2. The initial tree for the heuristic search was obtained automatically by applying the neighbour-joining and BioNJ algorithms to a matrix of pairwise distances estimated using the Tamura–Nei model, and then selecting the topology with superior log likelihood value; in total, 3414 positions were in the final dataset. The MLST-based phylogenetic tree of the concatenated sequences generated using the maximum likelihood method and Tamura–Nei model revealed three distinct lineages in the ESBL-producing STEC isolate strains. The majority of typed STs belonged to the clonal complex 10 (CC10), which showed amalgamated strains from HS, AM, ADW and NRW. The first clade represented an evolutionary history between two HS isolates assigned to ST117. The second clade, consisting of a mixture of HS, AM, and ADW, was assigned to ST23. The third clade represented a mixture of amalgamated strains from all matrices, predominantly assigned to ST10 and ST165 (Figure 3).

### 2.4. Detected ARGs in Isolated ESBL-Producing STEC

**Figure 4:**
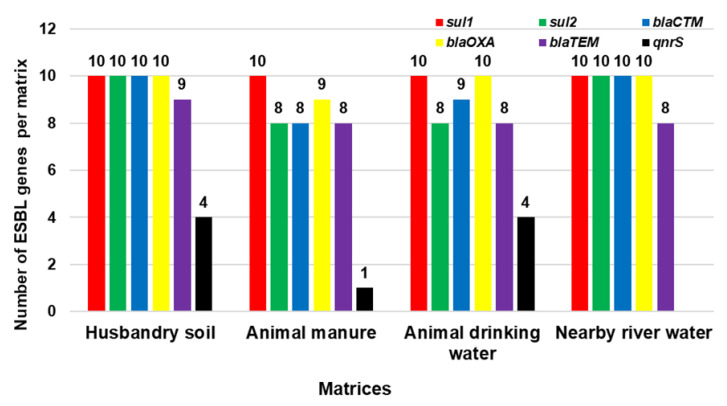
ARGs detected in ESBL-producing STEC isolates from sampled matrices.

For the selected ARGs (*sul*1, *sul*2, *bla_CTXM-M_*, *bla_OXA_*, *bla_TEM_*, and *qnrS*) in 40 randomly selected isolates, the most frequently detected gene for ESBL-producing STEC was *sul1*, which was detected in all 40 isolates (100%), followed by *bla*_OXA_ at 98% (*n* = 39), *bla*_CTX-M_ at 93% (*n* = 37), *sul*2 at 90% (*n* = 36), *bla_TEM_* at 83% (*n* = 33), and *qnrS* at 23% (*n* = 9). For HS, all 10 randomly selected isolates harboured *sul*1, *sul*2, *bla*_CTX-M_, and *bla*_OXA_; 9 isolates carried *bla*_TEM_ and 4 isolates carried *qnrS.* All 10 isolates from ADW harboured *sul*1 and *bla*_OXA_, with 9 isolates harbouring *bla*_CTX-M_, 8 isolates harbouring the *sul*2 and *bla*_TEM_ genes, and 4 isolates carrying *qnrS*. In NRW, *sul*1, *sul*2, *bla*_CTX-M_, and *bla*_OXA_ were detected in all 10 randomly selected isolates; 8 isolates carried *bla*_TEM_, while the *qnrS* gene was not detected in any of the isolates. For AM, *sul*1 was detected in all 10 isolates; 9 isolates carried *bla*_OXA_; 8 isolates carried *sul*2, *bla*_CTX-M_, and *bla*_TEM_; and only 1 isolate carried *qnrS.* The distribution of selected ARGs detected in this study is presented in Figure 4.

### 2.5. Detected Virulence Genes in Isolated ESBL-Producing STEC

**Figure 5:**
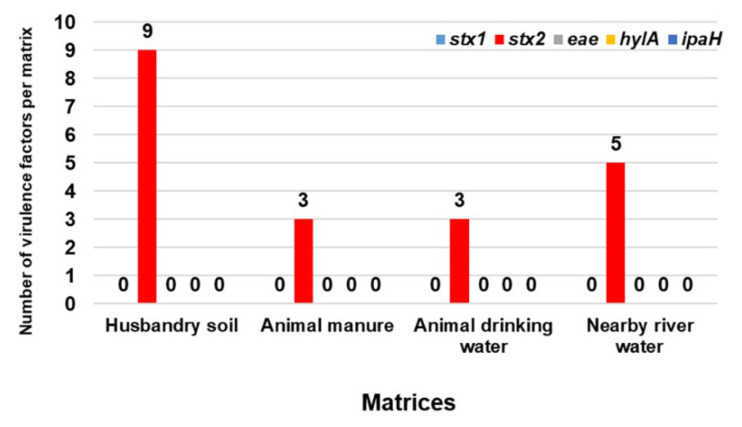
VFs detected in ESBL-producing STEC isolates from sampled matrices.

Of all the assessed virulence genes (*stx*1, *stx*2, *eae*, *hylA*, and *ipaH*), only the *stx*2 gene was detected across all the matrices (HS, AM, ADW, and NRW). In all 10 randomly selected ESBL-producing STEC isolates from each matrix, 9 (90%) HS isolates carried *stx*2. For AM and ADW, 3 isolates carried *stx*2 (30%), and 5 (50%) NRW isolates carried *stx*2. The distribution of virulence genes in ESBL-producing STEC isolates detected in all the matrices in this study is illustrated in Figure 5.

## 3. Discussion

The presence of ARB and ARGs in the livestock environment poses a threat to human health through exposure to the contaminated environment [12,42]. Runoff from the livestock farms can potentially contaminate the surface water, increasing the risk of foodborne illness as a result of the ingestion of contaminated water [43]. For this reason, it was crucial in this study to investigate ESBL-producing genes in STEC strains present in the livestock environment disseminating into the aquatic environment utilised by humans for drinking purposes. The current study isolated and characterised the ESBL-producing STEC in cattle associated environments by serotyping the STEC serogroups; typing STs; and detecting selected ARGs and the virulence genes harboured in HS, AM, ADW, and NRW samples, by using MLST-based phylogenetic analysis to establish relatedness. Currently, there has been little investigation into the prevalence of STEC and ESBL-producing STEC in cattle-associated environments that are potentially contaminated.

The prevalence of STEC and ESBL-producing STEC in this study has been reported from HS, AM, ADW, and NRW. Animal husbandry soil was shown to have the highest prevalence of STEC and ESBL-producing STEC, accounting for 94% and 83%, respectively. These high percentages might be due to the fact that the soil environment is considered as the major reservoir of STEC, particularly in agricultural soil; once STEC is in the soils, it can survive for days and up to months [44]. Furthermore, soil can also be contaminated with STEC as a result of manure application [44]. For ESBL-producing STEC, a high prevalence was observed as the soil is known to be a major reservoir of AR [45,46]. Animal manure can serve as a source of contamination, polluting both the soil and water [47]. In this present study, the prevalence of STEC in AM was 70.83%; for ESBL-producing STEC, it was 63.26%. The prolonged survival period of STEC and serogroup *E. coli* O157:H7 in manure being up to 21 months might clearly explain their prevalence, as also stated by previous investigators [48]. It is also important to note that manure is one of the matrices that is considered as a hotspot of many ARB and ARGs [49,50]. As for the ADW, the prevalence of STEC and ESBL-producing STEC was found to be higher (83.33% and 73.46%, respectively) compared to that of manure. Antibiotic residues can contaminate the environment, and due to accidental spillage of water fed to animals, they can contaminate the environment [51]. Furthermore, animal drinking water can influence the initiation and circulation of STEC within the livestock due to contamination of the animal drinking water troughs through direct or indirect faeces contamination from bedding material and dust in the troughs [52].

Water that is used by informal settlers and homeless people for basic needs such as drinking, and bathing was collected from the Bon Accord Dam. There are no reports of the prevalence of STEC and ESBL-producing STEC in this aquatic environment; thus, the current study reports for the first time the prevalence of STEC and ESBL-STEC in NRW to be 79.16% and 71.42%, respectively. Previous investigators have reported that water is also considered as the major carrier of STEC, which can persist for a long time and spread over long distances, escalating human exposure through direct contact; furthermore, aquatic environments are regarded as the main reservoir of AR [6,44,52,53].

Most STEC infections that cause major severe disease and outbreaks in humans belong to the big seven major serogroups, namely STEC O157, O26, O45, O103, O121, O111, and O145 [23]. The current study reports STEC serogroups in the livestock environment, namely in HS, AM, ADW, and NRW. The presence of STEC serogroups might differ because of regional variations or location [54,55]. Results of the present study agreed with the statement of these authors, as they demonstrated variations on the prevalence of the serogroups from aquatic environments surrounding the cattle farm under this investigation. The predominant serogroups in NRW were O45 at 80%, and 20% of all 10 randomly selected isolates were identified as O145. These results differ from those of [27], who only isolated and identified *E. coli* O157:H7, with a very low prevalence of 2.3% in water used for domestic purposes by residents of an informal settlement in Koster, a small farming town in North West Province of South Africa. The identified serogroups in the current study also differ from the identified serogroups in clinical settings.

MLST analysis was employed to establish the evolutionary relationship and relatedness between isolates in the livestock environment and the nearby aquatic environment. Internal fragments of the seven (7) housekeeping genes (*adk*, *fumC*, *gyrB*, *icd*, *mdh*, *purA*, and *recA*) were analysed to determine the relatedness of ESBL-producing STEC isolated from HS, AM, ADW, and NRW. Results of MLST analysis revealed four STs (ST10, ST23, ST165, and ST117) for the identified O45 and O145 serogroups of STEC belonging to the clonal complex (CC10), all of which were found to be ESBL-gene carriers. The STs within CC10 were found to have the following alleles: *adk* (*n* = 10), *fumC* (*n* = 11), *gyrB* (*n = 4*), *icd* (*n* = 8), *mdh* (*n* = 8), *purA* (*n* = 8), and *recA* (*n* = 2). An environmental study reported different STs from environmental water sources for the identified and characterised O157:H7 [32]. Different STs were assigned, namely ST10, ST11, and ST1204, in river water and runoff in the Gauteng Province [32]. Sequence type 10 in environmental waters was found to be similar to the dominant ST (ST10) in the current study. This included isolates from NRW (*n* = 10), HS (*n* = 6), ADW (*n* = 6), and AM (*n* = 5). The ST165, showing an intermix of two AM isolates, clustered together with ST10 isolates in the same clade. The second assigned ST was ST23, occurring in AM (*n* = 3), ADW (*n* = 3), and HS (*n* = 2). The ST23 clone has been previously identified with the ST10 clone from diseased cattle, which was found to be an ESBL-carrier in humans in France [56]. Moreover, ST117 was identified in the current study for two HS isolates that had been identified on a cattle farm isolated from cattle manure harbouring the *bla*_CTX-M-1_ gene in the Netherlands [57]. Molecular analysis revealed that AM, ADW, and NRW isolate sequences clustered together in one clade, suggesting that aquatic environments may be contaminated with ARGs of ESBL-producing STEC harbouring *stx2*. This finding might indicate that the dominant ST circulating from cattle environments to aquatic environments is ST10.

Our study indicated that ST10 was dominant, a sequence type known for its ubiquity in human faecal samples and in food samples. Moreover, the sequenced isolates in this study clustered together with the STs from the clinical settings. In the first clade (pink in Figure 3), isolates of HS belonging to ST117 clustered with STs (ST73, ST95, and ST131) that have been identified in clinics [33,34,58]. Although isolates had different STs, they clustered together; this might be due to the fact that they share five of the seven alleles. Such similarities in the antibiotic-resistant profiles of the isolates from different sources indicate similarities in antibiotic exposure histories and that all ESBLs are more closely related to one another [38,59]. The second clade (green in Figure 3) showed an intermix of isolates assigned to ST23 clustering with the dominant ST410 and ST648 from the clinics. Isolates of this study in the third clade (blue in Figure 3) also clustered with four of the STs (ST665, ST617, ST10, and ST744) that are dominant in clinical settings [58]. Despite the fact that they were not of the same STs, isolates of this study assigned as ST23 shared the same alleles with STs from clinical settings (*adk* = 6, *fumC* = 4, *gyrB* = 12, *icd* = 1, *mdh* = 20, and *recA* = 7). In the third clade (blue), our isolates that were identified as ST10 and ST165 (an intermix of HS, AM, ADW, and NRW isolates) clustered with ST 665, ST617, ST10, and ST744 identified in clinical settings by Mbelle et al. [58]. This occurrence might be due to the fact that these isolates from the present study shared the same alleles *(adk* = 10, *fumC* = 11, *gyrB* = 4, *icd* = 8, *mdh* = 8, *purA* = 8, and *recA* = 2). All the STs that were identified in our study showed close relatedness to those from the clinical settings.

For the selected ARGs (*sul*1, *sul*2, *bla*_CTX-M_, *bla*_OXA_, *bla*_TEM_, and *qnrS*) in this study and the 40 randomly selected isolates, the most frequently detected gene for ESBL-producing STEC was *sul*1, which occurred in all 40 isolates (100%), followed by *bla*_OXA_ at 97.5% (*n* = 39), *bl*a_CTX-M_ at 92.5% (*n* = 37), *sul*2 at 90% (*n* = 36), *bla*_TEM_ at 82.5% (*n* = 33), and *qnrS* at 22.5% (*n* = 9). The sulphonamide ARG *sul*1 occurred in all isolates (100%) and was the most frequently detected ARG, whereas *sul*2 was detected in 90% of all isolates. A high occurrence of *sul*1 and *sul*2 in environmental isolates has been reported widely [60]. The prevalence of *bla*_CTX-M_ was also high at 92.5%; CTX-M is known to be the predominant ESBL type in ARGs, and is most often reported as associated with resistance, and CTX-M type ESBLs are known to have originated from the environment [61,62]. The prevalence of *bla*_TEM_ was 82.5%; this may be associated with the resistance caused by TEM-type β-lactamase genes in Gram-negative bacteria [61]. A low prevalence of *qnrS* (22.5%) might be attributed to the fact that *qnr* genes are found on the same plasmids of ESBL and are reported have low level of resistance to fluoroquinolone in Enterobacteriaceae [63].

For all the matrices in the current study, HS harboured almost all the ESBL genes compared to other matrices; the distribution of ARGs in HS was as follows: *sul*1 (100%, *n* = 10), followed by *sul*2 (100%, *n* = 10), *bla*_CTX-M_ (100%, *n* = 10), *bla*_OXA_ (100%, *n* = 10), *bla*_TEM_ (90%, *n* = 9), and *qnrS* (40%, *n* = 4). The highest prevalence of ARGs was detected in soil. These findings corroborate those of previous investigators who stated that soil represents a natural reservoir of ARB, carrying a diverse set of known and unknown antibiotic resistance determinants [50]. The current study revealed that the ARGs present in AM isolates were *sul*1 (100%, *n* = 10), *sul*2 (80%, *n* = 8), *bla*_CTX-M_ (80%, *n* = 8), all of *bla*_OXA_ (90%, *n* = 9), *bla*_TEM_ (80%, *n* = 8), and *qnrS* (10%, *n* = 1). A high prevalence of ARGs detected in cattle manure might be due to the fact that manure is one of the matrices that is considered as the hotspot of bacteria carrying ARGs [50].

In ADW isolates, we observed *sul*1 (100%, *n* = 10), *sul*2 (80%, *n* = 8), *bla*_CTX-M_ (90%, *n* = 9), all of *bla*_OXA_ (100%, *n* = 10), *bla*_TEM_ (80%, *n* = 9), and *qnrS (*40%, *n* = 4). For NRW isolates, *sul*1 (100%, *n* = 10), *sul*2 (100%, *n* = 10), *bla*_CTX-M_ (100%, *n* = 10), and *bla*_OXA_ (100%, *n* = 10) were found to be the highest compared to *bla*_TEM_ (80%, *n* = 9), while *qnrS* was not detected. These results confirmed the detection of many ARGs in water as reported by previous investigators who reported that aquatic environments are known to be major reservoirs of ARB and ARGs as a result of the absorption of different pollutants [64,65]. The detected ARGs in the current study are similar to some ARGs identified from cattle manure samples, which are *bla*_CTX-M_ and *bla*_TEM_ from faecal samples belonging to the O157 serogroup [66]. Furthermore, *bla*_TEM_, *bla*_SHV_, and *bla*_CTX-M_ genes were detected in *E. coli* isolates from cattle manure and raw beef samples (Montso et al., 2019a); furthermore, in the present study, *bla*_TEM_ and *bla*_CTX-M_ were also detected. However, *sul*1 and *sul*2 were not targeted in either of the other studies. Most importantly, the target ESBL genes (namely *sul*1, *sul*2, *bla_CTX-M_*, *bla_OXA_*, *bla_TEM_*, and *qnrS*) detected in the current study are similar to some of those identified and detected in clinics [33,34,58,67,68]_._ This might due to the fact that ARGs present in clinical settings are thought to have originated from environmental bacteria and have been detected in genomes of environmental ARB [14].

Shiga toxin 1 (Stx1) and Shiga toxin 2 (Stx2) are the major virulence factors of STEC; one strain of STEC can produce both toxins or only one of the virulence factors [22,25]. In the present study, for the identified O45 and O145 serogroups, the virulence gene *stx*2 was identified in all the environmental samples. Animal husbandry soil carried 90% of *stx*2; in both AM and ADW, 30% of the isolates in each matrix carried *stx*2; for NRW, 50% of the isolates carried *stx*2. The current results contrast with those linked to virulence factors detected by Ndlovu et al. [69], who reported unspecified *stx* in 15%, *ipaH* in 31%, and *eae* in 8% of the river water samples collected from the Berg River. It has been suggested that the production of virulence factors depends on the serotype of STEC [70]. Shiga toxin 2 is associated with a higher risk of HUS, and it is 1000 times more toxic compared with *stx*1 [25,71]. Many countries continue to use antibiotics in livestock for growth promotion purposes, which can result in the introduction and dissemination of *stx*2 into the environment, which can potentially infect other bacteria [72]. Even though 20 isolates did not harbour Shiga toxin genes, we cannot state that the *stx* genes of these 20 isolates did not contain Shiga toxin genes, as they might be lost during isolation. Previous studies have reported the loss of *stx* genes upon isolation or subculture among STEC strains belonging to serogroups O2:H5, O26:H11, O73:H34, and O100:H32 [73].

## 4. Materials and Methods

### 4.1. Description of Study Area and Sample Collection

Animal husbandry soil (HS), animal manure (AM), animal drinking water (ADW) and nearby river water (NRW) were collected at the Tshwane University of Technology (TUT) Research Farm located in Honingnestkrans, near Bon Accord, in Pretoria North, Gauteng Province. A description of the study area and sample collection are detailed by Ramaite et al. [74]. Livestock-associated environmental samples were collected from October to December 2018, amounting to a total of 192 samples, including 48 samples from each source (HS, AM, ADW, and NRW).

### 4.2. Processing of Samples

Briefly, HS and AM samples were processed using the water-displacement method as described by Abia et al. [75] with slight modifications. About 300 g of HS or AM sample was aseptically transferred into a 1 L sterile Durham bottle containing 400 mL of 1× phosphate-buffered saline (PBS), 137 mM NaCl, 2.7 mM KCl, 8 mM Na_2_HPO_4_, and 2 mM KH_2_ until the 500 mL mark was reached. For both ADW and NRW, 100 mL of each sample was used directly from collected samples. For a full description of the processing of samples, refer to that described by Ramaite et al. [74].

### 4.3. Isolation of ESBL-Producing STEC

To isolate STEC and ESBL-producing STEC, 100 mL of each of the water samples or extractions (HS, AM, ADW, and NRW) were pre-enriched in 200 mL of tryptone soy broth (TSB, OXOID) (Thermo Scientific, Johannesburg, South Africa). Enriched broths were incubated at 42 °C for ±18 (Millipore Incubator X6310000, Merck Millipore, Johannesburg, South Africa), respectively. After incubation, a loopful of broths of the enriched cultures was spread on the CHROMagar™ STEC for isolation of STEC and on CHROMagar™ STEC supplemented with 0.57 g/L of CHROMagar™ ESBL for isolation of ESBL STEC (Media Mage (Pty) Ltd., Johannesburg, South Africa). *Escherichia coli* NCTC^®^ 11954 beta-lactamase-producing control strain and *Staphylococcus aureus* subsp. *aureus* Rosenbach (ATCC^®^ 43300™) were used as positive and negative quality controls, respectively (Thermo Scientific, Johannesburg, South Africa). Pure 100 presumptive colonies were randomly selected and placed separately in 2 mL of sterile TSB in Eppendorf tubes and centrifuged at 4000 rpm for 30 s to form bacterial pellets. Following incubation, the bacterial pellet was suspended in 20% glycerol and kept at −80 °C until further analysis.

For preliminary identification, 100 presumptive colonies were subjected to matrix-assisted laser desorption ionisation time-of-flight mass spectrometry (MALDI-TOF-MS) analysis at the University of Pretoria, Department of Microbiology and Plant Pathology (MALDI-TOF Diagnostic Service). The MALDI-TOF-MS procedure was carried out as described by Ramaite et al. [74].

### 4.4. Genomic DNA Extraction

Following the results of the MALDI-TOF analysis, confirmed ESBL-producing STEC isolates were thawed for DNA extraction. DNA was extracted from preserved cultures of ESBL-producing STEC isolates using InstaGene™ matrix according to the manufacturer’s instructions (Bio-Rad, Johannesburg, South Africa). The extracted genomic DNA (gDNA) was checked for quality and concentration using the NanoDrop™ 2000 spectrophotometer (Thermo Scientific, Johannesburg, South Africa). The extracted gDNA suspension was then kept at −80 °C until further use.

### 4.5. Molecular Analysis of ESBL-Producing STEC

#### 4.5.1. Identification of Serogroups of ESBL-Producing STEC

To identify the predominant STEC serogroups, all the ESBL-producing STEC isolates were further serotyped for their O serogroups using the conventional PCR. Each PCR was set up to detect the eight STEC O-groups, namely O26, O45, O103, O111, O113, O121, O145, and O157. The PCR amplification was carried out in a MiniAmp Plus thermal cycler (Thermo Fisher, Johannesburg, South Africa). Each PCR was performed in a total volume of 25 µL, consisting of 5 µL of extracted gDNA template, 0.5 µL of forward primer (10 µM) and 0.5 µL of reverse primer (10 µM), 12.5 µL of *Taq* 2X master mix, and 6.5 µL of nuclease-free water (Inqaba Biotechnical Industries, Pretoria, South Africa). The following conditions were used for PCR amplification: enzyme activation for 30 s at 95 °C, followed by 30 cycles of denaturation for 30 s at 95 °C, annealing at 57 °C for 1 min, extension for 1 min at 68 °C, and a final extension for 5 min at 68 °C. The expected band sizes of the PCR products were visualised by electrophoresis on a 1% agarose gel which was prepared in 1× TAE buffer and stained with 0.5 µL ethidium bromide. The electrophoresis was performed for 1 h at 100 V, and the amplicons in the gel were visualised under ultraviolet light using Syngene Gel documentation system (Vacutec, Johannesburg, South Africa). The primer pairs for the identification of O-serogroups are listed in Table 1.

#### 4.5.2. Molecular Typing of Selected ESBL-Producing *E. coli* Isolates

For multilocus sequence typing (MLST) of *E. coli* isolates, internal gene fragments of seven housekeeping genes (*adk*, *fumC*, *gyrB*, *icd*, *mdh*, *purA*, and *recA*) were sequenced as described by [77], using primers as listed in Table 1. The amplified PCR products were purified by mixing 10 µL of PCR reaction for sequencing preparation and 2.5 µL of ExoSAP Mix, consisting of 50 µL of Exonuclease I (NEB) and 200 µL of shrimp alkaline phosphatase. The reaction mixture was properly mixed and followed by incubation for 15 min at 37 °C. Following incubation, the mixture was heated for 15 min at 80 °C (Inqaba Biotechnical Industries, Johannesburg, South Africa). Purified PCR products were sequenced, as described by Ramaite et al. [74].

### 4.6. Multilocus Analysis

In brief, sequences were edited using Molecular Evolutionary Genetics Analysis (MEGA) software (Edit Menu in Alignment Explorer) and queried using the BLASTn algorithm (https://blast.ncbi.nlm.nih.gov/Blast.cgi, accessed on 13 February 2021). All the seven housekeeping genes used in the scheme (*adk*, *fumC*, *gyrB*, *icd*, *mdh*, *purA*, and *recA)* were concatenated by using MEGA X [81] and aligned by Muscle [82]. The evolutionary history was inferred by using the Maximum Likelihood method and the Tamura–Nei model [83]. The tree was drawn to scale, with branch lengths measured in the number of substitutions per site. The tree for the heuristic search was obtained automatically by applying neighbour-joining and BioNJ algorithms to a matrix of pairwise distances estimated using the Tamura–Nei model and then selecting the topology with superior log likelihood value. This analysis involved 40 nucleotide sequences. Codon positions included were 1st + 2nd + 3rd + Noncoding. There was a total of 3414 positions in the final dataset. Evolutionary analyses were conducted in MEGA X [81]. The inferred MLST-based phylogenetic tree was annotated using iTol [84], where STs, VF, and ARGs were allocated to their respective strains.

To gain insight into the relatedness between the isolates of ESBL-producing STEC in the present study and the predominant ST from the clinical settings, we inferred a phylogenetic tree using MLST sequences of our ESBL-producing STEC isolates against ST sequences retrieved with those published [33,34,58]. The isolates that were sequenced in the current study and from other published studies were aligned by muscle aligner in MEGA X [81] and analysed as described in the above section.

### 4.7. Genetic Detection of Selected ARGs

Selected ARGs, namely *sul1*, *sul2*, *bla_OXA_*, *bla_TEM_*, *bla_CTXM-M_*, and *qnrS*, were assessed. Amplification conditions for PCR were as described in the above section, and the primer annealing temperatures for *sul1*, *sul2*, *bla_OXA_*, *bla_TEM_*, *bla_CTXM-M_*, and *qnrS* were 56 °C, 60 °C, 60 °C, 53 °C, 53 °C, and 54 °C, respectively. The PCR products were visualised for expected band sizes, as stated above in the identification of STEC serogroups, using the primer pairs shown in Table 1.

### 4.8. Detection of Virulence Factors in ESBL-Producing STEC

Multiplex-PCR was used to screen samples for the presence of virulence genes (*stx*1, *stx*2, *eae*, *hylA*, and *ipaH*); the primer pairs are listed in Table 1. The amplification conditions for the PCR assay and for DNA amplification were as described in the above section for identification of STEC serogroups, with primer annealing for 1 min at 56 °C for *stx*1, *stx*2, and *eae* and for 1 min at 65 °C and 60 °C for *hylA* and *ipaH*, respectively.

### 4.9. Statistical Analysis

The prevalence of both STEC and ESBL-producing STEC was determined, STEC serotypes were identified, and the presence and distribution of VFs and ARGs genes were plotted using Microsoft Excel 2016 (Microsoft Corporation, Redmond, WA, USA). The prevalence of positive samples for each matrix was expressed as the percentage of positive samples from the total number of samples tested. Fischer’s exact test using formula in Microsoft Excel PowerPoint^®^ 2016 (Microsoft Corporation, Redmond, WA, USA) was used to evaluate the difference in the prevalence of ESBL-producing STEC between the four matrixes. Analysis was performed at the 95% (α = 0.05) confidence limit.

## 5. Conclusions

The present study investigated genetic characteristics of ESBL-producing STEC in cattle farm environments to track their dissemination from the livestock environment into the NRW. A considerable number of STEC non-O157 strains were isolated, and virulence genes and ARGs were observed in STEC non-O157 strains. The ST10 (*n* = 28) was more prevalent compared to other STs belonging to CC10, showing intermixed clades of ESBL-producing STEC isolated from different environments. The genetic profiles of isolates identified in livestock and aquatic environments were similar to those of clinical isolates. Cattle environments can be regarded as a reservoir of ESBL-producing bacteria that may spread to nearby aquatic environments. Therefore, continual surveillance of non-O157 and effective water quality control measures would be beneficial for controlling and preventing STEC diseases in communities that regularly make use of river water.

## Figures and Tables

**Table 1:** Primer pairs for identification of O-serogroups, housekeeping genes, ARGs, and virulence factor genes used in this study.

Gene Abbreviations	Prime Sequence (F—Forward, R—Reverse) from 5’ to 3’	Product Size (bp)	Annealing Temperature (°C)	References
O-serogroups				
O26	F: CAATGGGCG GAAATTTTAGA R: ATAATTTTCTCTGCCGTCGC	155	57	[76]
O45	F: TGCAGTAACCTGCACGGGCG R: AGCAGGCACAACAGCCACTACT	238	57	
O103	F: TTGGAGCGTTAACTGGACCT R: GCTCCCGAGCACGTATAAAG	321	57	
O113	F: TGCCATAATTCAGAGGGTGAC R: AACAAAGCTAA TTGTGGCCG	514	57	
O121	F: TCCAACAATTGGTCGTGAAA R: AGAAAG TGTGAAATGCCCGT	628	57	
O145	F: TTCATTGTTTTGCTTGCTCG R: GGCAAGCTTTGGAAATGAAA	750	57	
O157	F: TCGAGGTACCTGAATCTTTCCTTCTGT R: ACCAGTCTTGGTGCTGCTCTGACA	894	57	
Housekeeping genes				
adk	F: ATTCTGCTTGGCGCTCCGGG R: CCGTCAACTTTCGCGTATTT	582	54	[77]
fumC	F: TCACAGGTCGCCAGCGCTTC R: GTACGCAGCGAAAAAGATTC	806	54	
gyrB	F: TCGGCGACACGGATGACGGC R: ATCAGGCCTTCACGCGCATC	911	60	
icd	F: ATGGAAAGTAAAGTAGTTGTTCCGGCACA R: GGACGCAGCAGGATCTGTT	878	54	
mdh	F: ATGAAAGTCGCAGTCCTCGGCGCTGCTGGCGG R: TTAACGAACTCCTGCCCCAGAGCGATATCTTTCTT	932	60	
purA	F: CGCGCTGATGAAAGAGATGA R: CATACGGTAAGCCACGCAGA	816	54	
recA	F: CGCATTCGCTTTACCCTGACC R: TCTCGATCAGCTTCTCTTTT	780	58	
Antibiotic resistance genes				
*sul*1	F: CGCACCGGAAACATCGCTGCAC R: TGAAGTTCCGCCGCAAGGCTCG	163	56	[78]
*sul*2	F:TCCGGTGGAGGCCGGTATCTGG R: CGGGAATGCCATCTGCCTTGAG	191	60	
*bla*_CTX-M_	F: CGA TGTGCAGTACCAGTAA R: TTAGTGACCAGAATCAGCGG	585	60	[79]
*bla*_OXA_	F: TATCTACAGCAGCGCCAGTG R: CGCATCAAATGCCATAAGTG	199	53	
*bla*_TEM_	F: TACGATACGGGAGGGCTTAC R: TTCCTGTTTTTGCTCACCCA	716	53	
*qnrS*	F: GCAAGTTCATTGAACAGGGT R: TCTAAACCGTCGAGTTCGGCG	428	54	[78]
Virulence genes				
*stx*1	F: CAGTTAATGTGGTGGCGAAGG R: CACCAGACAATGTAACCGCTG	348	56	[71]
*stx*2	F: ATCCTATTCCCGGGAGTTTACG R: GCGTCATCGTATACACAGGAGC	584	56	
*Eae*	F: ATTACTGAGATTAAGGCTGA R: ATTTATTTGCAGCCCCCCAT	682	56	
*hlyA*	F: GCATCATCAAGCGTACGTTCC R: AATGAGCCAAGCTGGTTAAGCT	534	65	[80]
*ipaH*	F: CTCGGCACGTTTTAATAGTCTGG R: GTGGAGAGCTGAAGTTTCTCTGC	933	60	[29]

## Data Availability

Not applicable.

## Abbreviations and Acronyms

STEC	Shiga toxin-producing *Escherichia coli*
ESBL-STEC	Extended beta-lactamase- and Shiga toxin-producing *Escherichia coli*
HS	Husbandry soil
AM	Animal manure
ADW	Animal drinking water
NRW	Nearby river water
ARG	Antibiotic resistance gene

## References

[1] Cosgrove W.J., Loucks D.P. (2015). Water Management: Current and Future Challenges and Research Directions. Water Resour. Res..

[2] Butler D. (2017). Global Challenges: Water. Glob. Challenges.

[3] Pandey P.K., Kass P.H., Soupir M.L., Biswas S., Singh V.P. (2014). Contamination of Water Resources by Pathogenic Bacteria. AMB Express.

[4] Verlicchi P., Grillini V. (2020). Surface Water and Groundwater Quality in South Africa and Mozambique—Analysis of the Most Critical Pollutants for Drinking Purposes and Challenges in Water Treatment Selection. Waters.

[5] Moropeng R.C., Budeli P., Momba M.N.B. (2021). An Integrated Approach to Hygiene, Sanitation, and Storage Practices for Improving Microbial Quality of Drinking Water Treated at Point of Use: A Case Study in Makwane Village, South Africa. Int. J. Environ. Res. Public Health.

[6] Amarasiri M., Sano D., Suzuki S. (2019). Understanding Human Health Risks Caused by Antibiotic Resistant Bacteria (ARB) and Antibiotic Resistance Genes (ARG) in Water Environments: Current Knowledge and Questions to Be Answered. Crit. Rev. Environ. Sci. Technol..

[7] World Health Organization (2022). Antimicrobial Resistance Surveillance in Europe 2022–2020 Data.

[8] van den Honert M.S., Gouws P.A., Hoffman L.C. (2018). Importance and Implications of Antibiotic Resistance Development in Livestock and Wildlife Farming in South Africa: A Review. S. Afr. J. Anim. Sci..

[9] Van T.T.H., Yidana Z., Smooker P.M., Coloe P.J. (2020). Antibiotic Use in Food Animals Worldwide, with a Focus on Africa: Pluses and Minuses. J. Glob. Antimicrob. Resist..

[10] Ma Z., Lee S., Casey Jeong K. (2019). Mitigating Antibiotic Resistance at the Livestock-Environment Interface: A Review. J. Microbiol. Biotechnol..

[11] Aslam B., Wang W., Arshad M.I., Khurshid M., Muzammil S., Rasool M.H., Nisar M.A., Alvi R.F., Aslam M.A., Qamar M.U. (2018). Antibiotic Resistance: A Rundown of a Global Crisis. Infect. Drug Resist..

[12] Agga G.E., Cook K.L., Netthisinghe A.M.P., Gilfillen R.A., Woosley P.B., Sistani K.R. (2019). Persistence of Antibiotic Resistance Genes in Beef Cattle Backgrounding Environment over Two Years after Cessation of Operation. PLoS ONE.

[13] Yoshizawa N., Usui M., Fukuda A., Asai T., Higuchi H., Okamoto E., Seki K., Takada H., Tamura Y. (2020). Manure Compost Is a Potential Source of Tetracycline-Resistant Escherichia Coli and Tetracycline Resistance Genes in Japanese Farms. Antibiotics.

[14] Ekwanzala M.D., Dewar J.B., Kamika I., Momba M.N.B. (2018). Systematic Review in South Africa Reveals Antibiotic Resistance Genes Shared between Clinical and Environmental Settings. Infect. Drug Resist..

[15] Mateo-Sagasta J., Zadeh S.M., Turral H. Water Pollution from Agriculture: A Global Review. FAO IWMI 2017, Volume 35. http://www.fao.org/3/a-i7754e.pdf.

[16] WHO (2017). Global Priority List of Antibiotic-Resistant Batceria to Guide Research, Discovery, and Development of New Antibiotics.

[17] Franz E., Veenman C., van Hoek A.H.A.M., de Roda Husman A., Blaak H. (2015). Pathogenic Escherichia Coli Producing Extended-Spectrum β-Lactamases Isolated from Surface Water and Wastewater. Sci. Rep..

[18] Jang J., Hur H.-G., Sadowsky M.J., Byappanahalli M.N., Yan T., Ishii S. (2017). Environmental *Escherichia Coli*: Ecology and Public Health Implications—a Review. J. Appl. Microbiol..

[19] Lupindu A.M. (2018). Epidemiology of Shiga Toxin-Producing Escherichia Coli O157:H7 in Africa in Review. South. Afr. J. Infect. Dis..

[20] Etcheverría A.I., Padola N.L. (2013). Shiga Toxin-Producing Escherichia Coli: Factors Involved in Virulence and Cattle Colonization. Virulence.

[21] Kalule J.B., Keddy K.H., Nicol M.P. (2018). Characterisation of STEC and Other Diarrheic *E. Coli* Isolated on CHROMagar^TM^STEC at a Tertiary Referral Hospital, Cape Town. BMC Microbiol..

[22] Karama M., Cenci Goga B., Malahlela M., Smith, Keddy K., El-Ashram S., Lawan K. (2019). Kalake Virulence Characteristics and Antimicrobial Resistance Profiles of Shiga Toxin-Producing Escherichia Coli Isolates from Humans in South Africa: 2006–2013. Toxins.

[23] Mainga A.O., Cenci-Goga B.T., Malahlela M.N., Tshuma T., Kalake A., Karama M. (2018). Occurrence and Characterization of Seven Major Shiga Toxin-Producing Escherichia Coli Serotypes from Healthy Cattle on Cow-Calf Operations in South Africa. Zoonoses Public Health.

[24] Karama M., Mainga A.O., Cenci-Goga B.T., Malahlela M., El-Ashram S., Kalake A. (2019). Molecular Profiling and Antimicrobial Resistance of Shiga Toxin-Producing Escherichia Coli O26, O45, O103, O121, O145 and O157 Isolates from Cattle on Cow-Calf Operations in South Africa. Sci. Rep..

[25] Mathusa E.C., Chen Y., Enache E. (2010). Non-O157 Shiga Toxin—Producing Escherichia Coli in Foods. J. Food Prot..

[26] Monaghan A., Byrne B., Fanning S., Sweeney T., McDowell D., Bolton D.J. (2011). Serotypes and Virulence Profiles of Non-O157 Shiga Toxin-Producing Escherichia Coli Isolates from Bovine Farms. Appl. Environ. Microbiol..

[27] Ateba C.N., Mbewe M. (2011). Detection of Escherichia Coli O157:H7 Virulence Genes in Isolates from Beef, Pork, Water, Human and Animal Species in the Northwest Province, South Africa: Public Health Implications. Res. Microbiol..

[28] Majowicz S.E., Scallan E., Jones-Bitton A., Sargeant J.M., Stapleton J., Angulo F.J., Yeung D.H., Kirk M.D. (2014). Global Incidence of Human Shiga Toxin-Producing Escherichia Coli Infections and Deaths: A Systematic Review and Knowledge Synthesis. Foodborne Pathog. Dis..

[29] Tau N.P., Meidany P., Smith A.M., Sooka A., Keddy K.H., Group for Enteric and Meningeal Disease Surveillance in South Africa (2012). Escherichia Coli O104 Associated with Human Diarrhea, South Africa, 2004–2011. Emerg. Infect. Dis..

[30] Smith A.M., Tau N.P., Kalule B.J., Nicol M.P., McCulloch M., Jacobs C.A., McCarthy K.M., Ismail A., Allam M., Kleynhans J. (2019). Shiga Toxin-Producing Escherichia Coli O26:H11 Associated with a Cluster of Haemolytic Uraemic Syndrome Cases in South Africa, 2017. Access Microbiol..

[31] Croxen M.A., Law R.J., Scholz R., Keeney K.M., Wlodarska M., Finlay B.B. (2013). Recent Advances in Understanding Enteric Pathogenic Escherichia Coli. Clin. Microbiol. Rev..

[32] Bolukaoto J.Y., Kock M.M., Strydom K.A., Mbelle N.M., Ehlers M.M. (2019). Molecular Characteristics and Genotypic Diversity of Enterohaemorrhagic Escherichia Coli O157:H7 Isolates in Gauteng Region, South Africa. Sci. Total Environ..

[33] Peirano G., Greune C., Pitout J. (2011). Characteristics of Infections Caused by Extended-Spectrum β-Lactamase-Producing Escherichia Coli FromC Community Hospitals in South Africa. Diagn. Microbiol. Infect. Dis..

[34] Gqunta K., Govender S. (2015). Characterization of ESBL-Producing Escherichia Coli ST131 Isolates from Port Elizabeth. Diagn. Microbiol. Infect. Dis..

[35] Dantas Palmeira J., Ferreira H.M.N. (2020). Extended-Spectrum Beta-Lactamase (ESBL)-Producing Enterobacteriaceae in Cattle Production – a Threat around the World. Heliyon.

[36] Abayneh M., Tesfaw G., Abdissa A. (2018). Isolation of Extended-Spectrum *β*-Lactamase- (ESBL-) Producing *Escherichia Coli* and *Klebsiella Pneumoniae* from Patients with Community-Onset Urinary Tract Infections in Jimma University Specialized Hospital, Southwest Ethiopia. Can. J. Infect. Dis. Med. Microbiol..

[37] Doi Y., Park Y.S., Rivera J.I., Adams-Haduch J.M., Hingwe A., Sordillo E.M., Lewis J.S., Howard W.J., Johnson L.E., Polsky B. (2013). Community-Associated Extended-Spectrum β-Lactamase-Producing Escherichia Coli Infection in the United States. Clin. Infect. Dis..

[38] Montso K.P., Dlamini S.B., Kumar A., Ateba C.N. (2019). Antimicrobial Resistance Factors of Extended-Spectrum Beta-Lactamases Producing Escherichia Coli and Klebsiella Pneumoniae Isolated from Cattle Farms and Raw Beef in North-West Province, South Africa. Biomed Res. Int..

[39] Iwu C.J., Iweriebor B.C., Obi L.C., Okoh A.I. (2016). Occurrence of Non-O157 Shiga Toxin-Producing Escherichia Coli in Two Commercial Swine Farms in the Eastern Cape Province, South Africa. Comp. Immunol. Microbiol. Infect. Dis..

[40] Montso P.K., Mlambo V., Ateba C.N. (2019). The First Isolation and Molecular Characterization of Shiga Toxin-Producing Virulent Multi-Drug Resistant Atypical Enteropathogenic Escherichia Coli O177 Serogroup From South African Cattle. Front. Cell. Infect. Microbiol..

[41] Oporto B., Ocejo M., Alkorta M., Marimón J.M., Montes M., Hurtado A. (2019). Zoonotic Approach to Shiga Toxin-Producing Escherichia Coli: Integrated Analysis of Virulence and Antimicrobial Resistance in Ruminants and Humans. Epidemiol. Infect..

[42] Graham D.W., Bergeron G., Bourassa M.W., Dickson J., Gomes F., Howe A., Kahn L.H., Morley P.S., Scott H.M., Simjee S. (2019). Complexities in Understanding Antimicrobial Resistance across Domesticated Animal, Human, and Environmental Systems. Ann. N. Y. Acad. Sci..

[43] Tanaro J.D., Piaggio M.C., Galli L., Gasparovic A.M.C., Procura F., Molina D.A., Vitón M., Zolezzi G., Rivas M. (2014). Prevalence of Escherichia Coli O157:H7 in Surface Water near Cattle Feedlots. Foodborne Pathog. Dis..

[44] Ma J., Mark Ibekwe A., Crowley D.E., Yang C.H. (2014). Persistence of Escherichia Coli O157 and Non-O157 Strains in Agricultural Soils. Sci. Total Environ..

[45] D/’Costa V.M., King C.E., Kalan L., Morar M., Sung W.W.L., Schwarz C., Froese D., Zazula G., Calmels F., Debruyne R. (2011). Antibiotic Resistance Is Ancient. Nature.

[46] Zheng B., Huang C., Xu H., Guo L., Zhang J., Wang X., Jiang X., Yu X., Jin L., Li X. (2017). Occurrence and Genomic Characterization of ESBL-Producing, MCR-1-Harboring Escherichia Coli in Farming Soil. Front. Microbiol..

[47] Manyi-Loh C.E., Mamphweli S.N., Meyer E.L., Makaka G., Simon M., Okoh A.I. (2016). An Overview of the Control of Bacterial Pathogens in Cattle Manure. Int. J. Environ. Res. Public Health.

[48] Callaway T.R., Edrington T.S., Loneragan G.H., Carr M.A., Nisbet D.J. (2013). Shiga Toxin-Producing Escherichia Coli (STEC) Ecology in Cattle and Management Based Options for Reducing Fecal Shedding. Agric. food Anal. Bacteriol..

[49] Udikovic-Kolic N., Wichmann F., Broderick N.A., Handelsman J. (2014). Bloom of Resident Antibiotic-Resistant Bacteria in Soil Following Manure Fertilization. Proc. Natl. Acad. Sci. USA.

[50] Thanner S., Drissner D., Walsh F. (2016). Antimicrobial Resistance in Agriculture. MBio.

[51] Chee-Sanford J.C., Mackie R.I., Koike S., Krapac I., Maxwell S., Lin Y.F., Aminov R.I. (2009). Fate and Transport of Antibiotic Residues and Antibiotic Resistance Genes. J. Environ. Qual..

[52] Farrokh C., Jordan K., Auvray F., Glass K., Oppegaard H., Raynaud S., Thevenot D., Condron R., De Reu K., Govaris A. (2012). International Journal of Food Microbiology Review of Shiga-Toxin-Producing Escherichia Coli (STEC ) and Their Signi Fi Cance in Dairy Production. Int. J. Food Microbiol..

[53] Conrad C., Stanford K., Mcallister T., Thomas J., Reuter T. (2016). Shiga Toxin-Producing Escherichia Coli and Current Trends in Diagnostics. Anim. Front..

[54] Paton J.C., Paton A.W. (1998). Pathogenesis and Diagnosis of Shiga Toxin-Producing Escherichia Coli Infections. Clin. Microbiol. Rev..

[55] Stanford K., Johnson R.P., Alexander T.W., McAllister T.A., Reuter T. (2016). Influence of Season and Feedlot Location on Prevalence and Virulence Factors of Seven Serogroups of Escherichia Coli in Feces of Western-Canadian Slaughter Cattle. PLoS ONE.

[56] Dahmen S., Métayer V., Gay E., Madec J.-Y., Haenni M. (2013). Characterization of Extended-Spectrum Beta-Lactamase (ESBL)-Carrying Plasmids and Clones of Enterobacteriaceae Causing Cattle Mastitis in France. Vet. Microbiol..

[57] Hordijk J., Mevius D.J., Kant A., Bos M.E.H., Graveland H., Bosman A.B., Hartskeerl C.M., Heederik D.J.J., Wagenaar J.A. (2013). Within-Farm Dynamics of ESBL/AmpC-Producing Escherichia Coli in Veal Calves: A Longitudinal Approach. J. Antimicrob. Chemother..

[58] Mbelle N.M., Feldman C., Osei Sekyere J., Maningi N.E., Modipane L., Essack S.Y. (2019). The Resistome, Mobilome, Virulome and Phylogenomics of Multidrug-Resistant Escherichia Coli Clinical Isolates from Pretoria, South Africa. Sci. Rep..

[59] Bradford P.A. (2001). Extended-Spectrum Beta-Lactamases in the 21st Century: Characterization, Epidemiology, and Detection of This Important Resistance Threat. Clin. Microbiol. Rev..

[60] Berglund B. (2015). Environmental Dissemination of Antibiotic Resistance Genes and Correlation to Anthropogenic Contamination with Antibiotics. Infect. Ecol. Epidemiol..

[61] Rahman S., Ali T., Ali I., Khan N.A., Han B., Gao J. (2018). The Growing Genetic and Functional Diversity of Extended Spectrum Beta-Lactamases. BioMed Res. Int..

[62] Lee S., Mir R.A., Park S.H., Kim D., Kim H.Y., Boughton R.K., Morris J.G., Jeong K.C. (2020). Prevalence of Extended-Spectrum β-Lactamases in the Local Farm Environment and Livestock: Challenges to Mitigate Antimicrobial Resistance. Crit. Rev. Microbiol..

[63] Salah F.D., Soubeiga S.T., Ouattara A.K., Sadji A.Y., Metuor-Dabire A., Obiri-Yeboah D., Banla-Kere A., Karou S., Simpore J. (2019). Distribution of Quinolone Resistance Gene (Qnr) in ESBL-Producing Escherichia Coli and Klebsiella Spp. in Lomé, Togo. Antimicrob. Resist. Infect. Control.

[64] Manyi-Loh C., Mamphweli S., Meyer E., Okoh A. (2018). Antibiotic Use in Agriculture and Its Consequential Resistance in Environmental Sources: Potential Public Health Implications. Molecules.

[65] Su S., Li C., Yang J., Xu Q., Qiu Z., Xue B., Wang S., Zhao C., Xiao Z., Wang J. (2020). Distribution of Antibiotic Resistance Genes in Three Different Natural Water Bodies-a Lake, River and Sea. Int. J. Environ. Res. Public Health.

[66] Iweriebor B.C., Iwu C.J., Obi L.C., Nwodo U.U., Okoh A.I. (2015). Multiple Antibiotic Resistances among Shiga Toxin Producing Escherichia Coli O157 in Feces of Dairy Cattle Farms in Eastern Cape of South Africa. BMC Microbiol..

[67] Ehlers M.M., Veldsman C., Makgotlho E.P., Dove M.G., Hoosen A.A., Kock M.M. (2009). Detection of BlaSHV, BlaTEM and BlaCTX-M Antibiotic Resistance Genes in Randomly Selected Bacterial Pathogens from the Steve Biko Academic Hospital. FEMS Immunol. Med. Microbiol..

[68] Osei Sekyere J., Maningi N.E., Modipane L., Mbelle N.M. (2020). Emergence of *mcr-9.1* in Extended-Spectrum-β-Lactamase-Producing Clinical *Enterobacteriaceae* in Pretoria, South Africa: Global Evolutionary Phylogenomics, Resistome, and Mobilome. mSystems.

[69] Ndlovu T., Le Roux M., Khan W., Khan S. (2015). Co-Detection of Virulent Escherichia Coli Genes in Surface Water Sources. PLoS ONE.

[70] Kobayashi N., Lee K., Yamazaki A., Saito S., Furukawa I., Kono T., Maeda E., Isobe J., Sugita-Konishi Y., Hara-Kudo Y. (2013). Virulence Gene Profiles and Population Genetic Analysis for Exploration of Pathogenic Serogroups of Shiga Toxin-Producing Genus-Species Escherichia Coli. J. Clin. Microbiol..

[71] Dong H.J., Lee S., Kim W., An J.U., Kim J., Kim D. (2017). Prevalence, Virulence Potential, and Pulsed - Field Gel Electrophoresis Profiling of Shiga Toxin - Producing Escherichia Coli Strains from Cattle. Gut Pathog..

[72] Griffin P.M., Karmali M.A. (2017). Emerging Public Health Challenges of Shiga Toxin-Producing Escherichia Coli Related to Changes in the Pathogen, the Population, and the Environment. Clin. Infect. Dis..

[73] Schmidt H., Scheef J., Huppertz H.I., Frosch M., Karch H. (1999). Escherichia Coli O157:H7 and O157:H(-) Strains That Do Not Produce Shiga Toxin: Phenotypic and Genetic Characterization of Isolates Associated with Diarrhea and Hemolytic-Uremic Syndrome. J. Clin. Microbiol..

[74] Ramaite K., Ekwanzala M.D., Dewar J.B., Momba M.N.B. (2021). Human-Associated Methicillin-Resistant Staphylococcus Aureus Clonal Complex 80 Isolated from Cattle and Aquatic Environments. Antibiotics.

[75] Abia L.K.A., Ubomba-Jaswa E., Ssemakalu C.C., Momba M.N.B. (2015). Development of a Rapid Approach for the Enumeration of Escherichia Coli in Riverbed Sediment: Case Study, the Apies River, South Africa. J. Soils Sediments.

[76] DebRoy C., Roberts E., Valadez A.M., Dudley E.G., Cutter C.N. (2011). Detection of Shiga Toxin–Producing Escherichia Coli O26, O45, O103, O111, O113, O121, O145, and O157 Serogroups by Multiplex Polymerase Chain Reaction of the Wzx Gene of the O-Antigen Gene Cluster. Foodborne Pathog. Dis..

[77] Wirth T., Falush D., Lan R., Colles F., Mensa P., Wieler L.H., Karch H., Reeves P.R., Maiden M.C.J., Ochman H. (2006). Sex and Virulence in Escherichia Coli: An Evolutionary Perspective. Mol. Microbiol..

[78] Mu Q., Li J., Sun Y., Mao D., Wang Q., Luo Y. (2015). Occurrence of Sulfonamide-, Tetracycline-, Plasmid-Mediated Quinolone- and Macrolide-Resistance Genes in Livestock Feedlots in Northern China. Environ. Sci. Pollut. Res..

[79] Kennedy C.A., Fanning S., Karczmarczyk M., Byrne B., Monaghan Á., Bolton D., Sweeney T. (2017). Characterizing the Multidrug Resistance of Non-O157 Shiga Toxin-Producing Escherichia Coli Isolates from Cattle Farms and Abattoirs. Microb. Drug Resist..

[80] Bannon J., Melebari M., Jordao C., Leon-Velarde C.G., Warriner K. (2016). Incidence of Top 6 Shiga Toxigenic Escherichia Coli within Two Ontario Beef Processing Facilities: Challenges in Screening and Confirmation Testing. AIMS Microbiol..

[81] Kumar S., Stecher G., Li M., Knyaz C., Tamura K. (2018). MEGA X: Molecular Evolutionary Genetics Analysis across Computing Platforms. Mol. Biol. Evol..

[82] Edgar R.C. (2004). MUSCLE: A Multiple Sequence Alignment Method with Reduced Time and Space Complexity. BMC Bioinformatics.

[83] Tamura K., Nei M. (1993). Estimation of the Number of Nucleotide Substitutions in the Control Region of Mitochondrial DNA in Humans and Chimpanzees. Mol. Biol. Evol..

[84] Letunic I., Bork P. (2016). Interactive Tree of Life (ITOL) v3: An Online Tool for the Display and Annotation of Phylogenetic and Other Trees. Nucleic Acids Res..

